# Multivariate Spectroscopic Analysis of Protein Secondary Structures in Gingival Crevicular Fluid: Insights from FTIR Amide III Band Across Oral Disease Stages

**DOI:** 10.3390/ijms26104693

**Published:** 2025-05-14

**Authors:** Pavel Seredin, Tatiana Litvinova, Yuri Ippolitov, Dmitry Goloshchapov, Yaroslav Peshkov, Boknam Chae, Raul O. Freitas, Francisco C. B. Maia

**Affiliations:** 1 Department of Solid-State Physics and Nanostructures, Voronezh State University, 394018 Voronezh, Russia; 2 Department of Pediatric Dentistry with Orthodontia, Voronezh State Medical University, 394006 Voronezh, Russia; 3 Pohang Accelerator Laboratory, Beamline Research Division, Pohang 37673, Republic of Korea; 4 Brazilian Synchrotron Light Laboratory (LNLS), Brazilian Center for Research in Energy and Materials (CNPEM), Campinas 13083-970, Brazil

**Keywords:** gingival crevicular fluid, FTIR, Amide III, proteins secondary structure, dental caries, periodontal diseases, oral diseases, multivariate analysis

## Abstract

This study applies multivariate data analysis to deconvolute the spectral profiles of the Amide III region in the infrared spectra of gingival crevicular fluid (GCF). This reveals the impact of major oral diseases, such as dental caries and periodontal diseases, on the transformation of the secondary structure of GCF proteins. A two-stage analytical approach was employed: first, principal component analysis (PCA) was performed to establish the main factors of variation in the data, followed by pairwise comparisons of the samples based on the results of the Amide III profile deconvolution. The analysis also accounted for comorbidities, such as oncological and gastrointestinal diseases. This approach allowed for the identification of subtle differences in the composition and conformation of the secondary structure of GCF proteins while accounting for the superposition of multiple influencing factors. This methodology was effective in identifying biomarkers of oral diseases in GCF. For the first time, it has been demonstrated that the relative content of the β-sheet-associated component in the spectral profile of the secondary structure element of the protein fraction of GCF serves as a statistically significant marker for dental caries, regardless of the presence or absence of other diseases. Additionally, a significant decrease in the relative content of α-helix structures was observed in GCF from patients with oncological diseases. The changes in the spectral profile of the Amide III band of GCF identified in this study have not been previously detected using molecular spectroscopy, correlated with the secondary structure of proteins, or analyzed using multivariate analysis methods.

## 1. Introduction

Gingival crevicular fluid (GCF) has been the focus of extensive research since the mid-20th century [1]. In recent years, its clinical potential has gained increasing attention, particularly as a promising medium for early detection of oral diseases [2,3]. One of the main advantages of utilizing GCF is its collection method, which is non-traumatic, minimally invasive, and relatively simple to perform [4]. As GCF seeps from ruptured microcapillaries in the gums, its molecular composition provides insight into various local pathological processes occurring in the periodontium [5]. The soft tissues of the periodontium serve protective and supportive functions in cases of infection [6]. Periodontal disease typically begins with gingivitis, characterized by gum inflammation, and can progress to periodontitis, which affects deeper structures, such as the periodontal ligament and alveolar bone. Dental caries and periodontal diseases are the most prevalent non-communicable diseases in humans, leading to a significant negative impact on the quality of life [7,8]. The oral microbiome plays a crucial role in the development of both conditions [9,10]. A shift in the oral microbiome, along with the resulting inflammation of the periodontal tissues, influences the composition of the exudate from the soft tissues of the gums, particularly in its protein fraction.

GCF is an inflammatory exudate originating from periodontal tissues and may contain a diverse array of substances that can serve as potential biomarkers. These include cytokines, antibodies, enzymes, components of blood serum, and degradation products such as cells, connective tissue elements, epithelial elements, and microbial flora [11]. Furthermore, as bacterial metabolites in GCF can enter the bloodstream, an immune response may occur in areas distant from the oral cavity [1]. The proteomic profile of GCF is characterized by proteins that can act as molecular markers of tissue damage [3]. Numerous proteins have been identified in GCF, with the most frequently cited being actin, keratins, histones, and annexins [12]. Recent studies have also identified LIGHT as a potential target for monitoring the dysregulation of the inflammatory response associated with the early destruction of periodontal tissues [13]. LIGHT is a secreted protein of the TNF superfamily that interacts with the lymphotoxin β receptor (LTβR) [13].

As previously demonstrated, changes in the molecular profile of GCF during the progression of various pathologies can be influenced by individual characteristics such as age, sex, and the patient’s medical history [3,14]. Moreover, comorbidities play a significant role. While primary oral pathologies such as caries and periodontal diseases occur independently, certain associations between them can be observed [3]. Therefore, a more comprehensive understanding of the nature and dynamics of this interrelationship necessitates the development of innovative approaches to analyze molecular changes in GCF resulting from multifactorial inflammatory diseases of the oral cavity, all while considering comorbidities.

Various approaches are commonly employed to characterize the GCF, such as biochemical or mass spectrometric analysis [15]. However, these methods tend to be time-consuming, labor-intensive, and require complex procedures for sample preparation.

At the same time, molecular methods such as Raman and Fourier Transform Infrared (FTIR) spectroscopy have proven to be highly effective tools for rapid diagnostics. These methods are employed across various fields of research, including chemistry, biochemistry, pharmacology, materials science, forensic analysis, and the examination of biochemical and structural changes in biological fluid samples [16,17]. These tools do not require complex sample preparation procedures. Infrared (IR) spectroscopy has been consistently demonstrated to be a straightforward and non-invasive method for monitoring chemical and structural changes in GCF samples [18].

Typically, when studying changes in the composition and structure of proteins, the Amide I and II bands in the FTIR spectrum, located in the range of 1700–1500 cm^−1^, are analyzed. These bands are primarily associated with C=O stretching vibrations (approximately 80%) and C-N stretching and N-H bending vibrations [19]. A detailed analysis of the spectral profiles of the Amide I and II bands in GCF can provide valuable insights into changes in the protein fraction related to transformations in the secondary structure of proteins, including the formation of amyloid aggregates induced by mechanical stress associated with orthodontic treatments [4]. A mathematical assessment of shifts in the secondary structure of proteins can be derived from the deconvolution of the full spectral profile of Amide I and II [20]. This assessment reflects the cariogenic situation in the oral cavity, taking into account medication intake. However, strong interference from water vibrations and their overlap with modes corresponding to various secondary structures in the Amide I and II regions complicate the interpretation of the results obtained. Therefore, analyzing the alternative Amide III IR band, which is formed by the overlapping N-H and C-N vibrations, appears promising, as it is also quite sensitive to the secondary structure of proteins [21,22]. Despite the fact that the intensity of this band is significantly weaker than that of the Amide I and II bands, its analysis offers a key advantage: there are no water vibrations in the Amide III region [21,23]. A precise examination of the FTIR spectral profile of the Amide III band is a powerful tool for establishing conformational changes in various secondary structural elements of proteins in GCF, such as α-helix, β-sheet, β-turn, and random coils.

It is important to note that collecting a substantial number of samples to identify the influence of various factors on the molecular composition of GCF presents significant challenges, particularly concerning ethical and clinical considerations. In this context, it is essential to utilize tools for multivariate statistical analysis and modeling that are suitable for small datasets. Multivariate statistical analysis of spectral data, particularly principal component analysis (PCA) with result visualization [14,24,25,26,27,28,29,30], can effectively reveal hidden structures within the data, identify sample clusters, and highlight key factors contributing to variations in spectra. These analytical methods can be successfully implemented using packages in the R (version 4.3.2) programming language, which facilitates the reproducibility of the results [31]. In this field, the use of the aforementioned packages is infrequent, which underscores the novelty of our work and establishes a trend toward enhancing the reproducibility of the research on the molecular FTIR spectra of GCF.

In our previous work [14], we utilized advanced multivariate methods to analyze synchrotron molecular spectroscopy data of GCF samples. This analysis revealed associations between the stages of dental caries development and periodontal pathologies, as well as additional factors reflecting the patient’s personalized clinical profile, including demographic characteristics and their comorbidities. These findings support the hypothesis that pathological processes in the human body may indirectly influence the spatial and conformational structures of proteins in GCF [14].

A review of the available literature revealed a lack of data on the characteristics of the secondary structure of GCF proteins in relation to the development of oral diseases. Therefore, the primary objective of this study was to conduct a multivariate analysis of Amide III spectral profiles to identify the main factors driving the variation in FTIR spectra and to interpret the transformation of the secondary structure of GCF proteins in the context of dental caries and periodontal diseases among patients with various comorbidities.

## 2. Results and Discussion

### Multivariate Analysis to Uncover Hidden Features and Key Factors of Variation in Spectral Data

**Figure 1:**
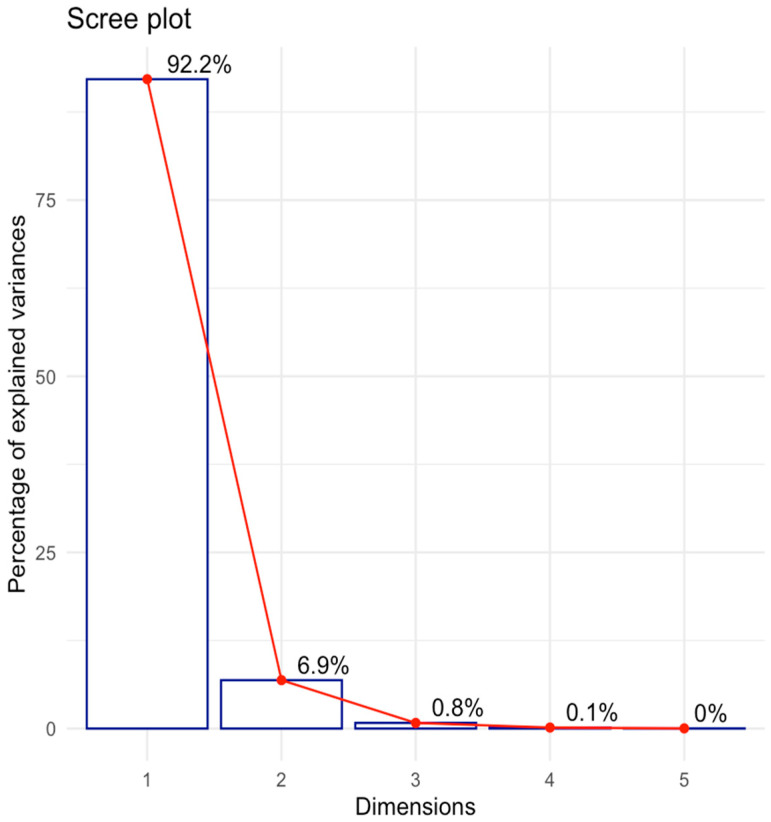
A scree plot.

In the initial stage of the analysis, the number of principal components was determined. Several approaches can be used to address this issue. According to the Kaiser criterion, only components with eigenvalues greater than 1 should be considered for further analysis [32]. In our study, the first two components met this criterion, collectively accounting for 99.02% of the variation. The percentage of variation explained by the principal components in the spectra was visualized using a scree plot (see Figure 1).

A common empirical practice for selecting the number of components is the Elbow rule. According to this rule, components that exhibit a sharp decrease in the percentage of explained variance on the scree plot (Figure 1) are excluded from further analysis [32].

**Figure 2:**
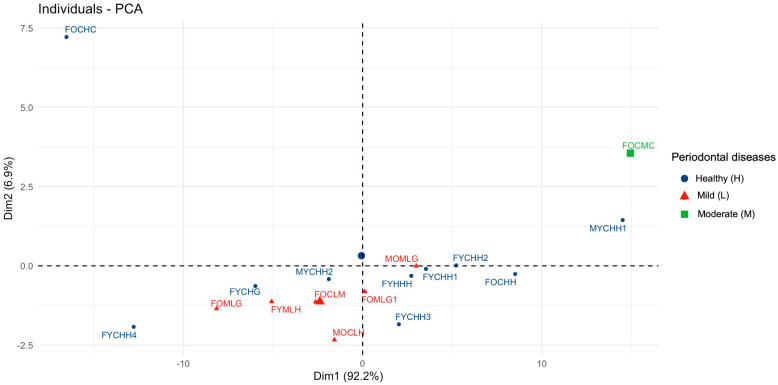
Visualization of PCA (PC1 and PC2). The leading factor of variation is “**Periodontal diseases**”.

For further analysis, we selected the first two components based on the criteria described above. Figure 2 illustrates the visualization of the PCA results using the R package factoextra, with the centers of the clusters marked in their corresponding colors.

This visualization enables us to identify distinct groups within the spectral data. We observe a contrast along the first component between healthy individuals (blue cluster) and those with periodontal disease. Among the latter, we can further distinguish groups with mild periodontitis (red cluster) and moderate periodontitis (green cluster), the latter, which consists of a single sample in this case. As illustrated in the figure, the green cluster is distinctly separated from the other two clusters, while the samples from the blue and red clusters exhibit a tendency to overlap.

The results of the *dimdesc* function from the R package FactoMineR reveal a relationship between the factor “**Periodontal diseases**” and the first principal component (PC1). However, this relationship is only significant for the “***Moderate periodontitis***” level of this factor, as indicated by the *t*-test (Estimate = 12.41, *p*-value = 0.048), which aligns with the visual interpretation of the data. It is important to note that the influence of the “**Periodontal diseases**” factor on the integral intensities in the FTIR spectra of GCF was demonstrated in our previous work [14], further reinforcing the findings from this analysis.

The second principal component (PC2) is strongly associated with the factor “**Comorbidities**”, as evidenced by a significant one-way ANOVA result (*p*-value = 0.0002), with an R^2^ value of 0.78. This indicates that 78% of the variation in spectral data can be explained by this factor [33]. Furthermore, the post-hoc *t*-test results further support the association of PC2 with the “***Oncological disease***” level of “**Comorbidities**” factor (Estimate = 4.67, *p*-value < 0.0001).

By examining the relationships between the levels of the studied factors and the quantitative variables (integral intensity values), we identified a statistically significant association (v.test, *p* < 0.05) between the “***Moderate Periodontitis***” level of the “**Periodontal diseases**” factor and the integral intensity values within the 1250–1210 cm^−1^ wave number range. Notably, higher integral intensity values in the FTIR spectral region were characteristic of the GCF sample obtained from a patient with moderate periodontitis.

In GCF samples obtained from patients with oncological diseases, significantly higher integral intensity values were observed within the 1209–1201 cm⁻^1^ spectral range (v.test, *p* < 0.05).

The Euclidean distances between the samples based on their factor levels, calculated in a two-dimensional space using the **dist** function from the FactoMineR package (see Section 3), are listed in Table 1.

The results indicate that the most significant differences (reflected by the highest Euclidean distance values) are observed between various levels of the factor “**Periodontal diseases**”. The “***Moderate Periodontitis***” level of this factor is the most distant from the other levels within this factor (bold numbers in Table 1).

Similarly, notable differences are observed within the “**Comorbidities**” factor levels, with “**Oncological disease**” showing the greatest distance from other levels. In contrast, the smallest distances are observed between the “***Gastrointestinal diseases***” and “***Multiple pathologies***” levels of this factor, which is consistent with the results of visualizations and statistical tests.

Regarding demographic characteristics, the data (see Table 1) reveal only minor differences between samples across levels of the “**Age**” factor, while the distance associated with the levels of “**Gender**” factor is more substantial. Additionally, minimal differences are noted between the levels of the “**Caries**” factor. This finding suggests the need for further research using alternative analytical methods to better identify spectral features linked to the presence or absence of caries, especially given the dominant influence of “**Periodontal diseases**” and “**Comorbidities**”.

To detect subtle differences in the secondary structure of the FTIR spectra related to periodontal condition and the presence or absence of caries, while considering the established relationships among various influencing factors, we selected the following samples from our spectral dataset (see Section 3.2. Dataset Description): FYHHH, FOCHH, FOCMC, and FOMLG. All selected samples were homogeneous with respect to sex and age, thereby eliminating potential confounding effects due to demographic variation (Table 2).

**Figure 3:**
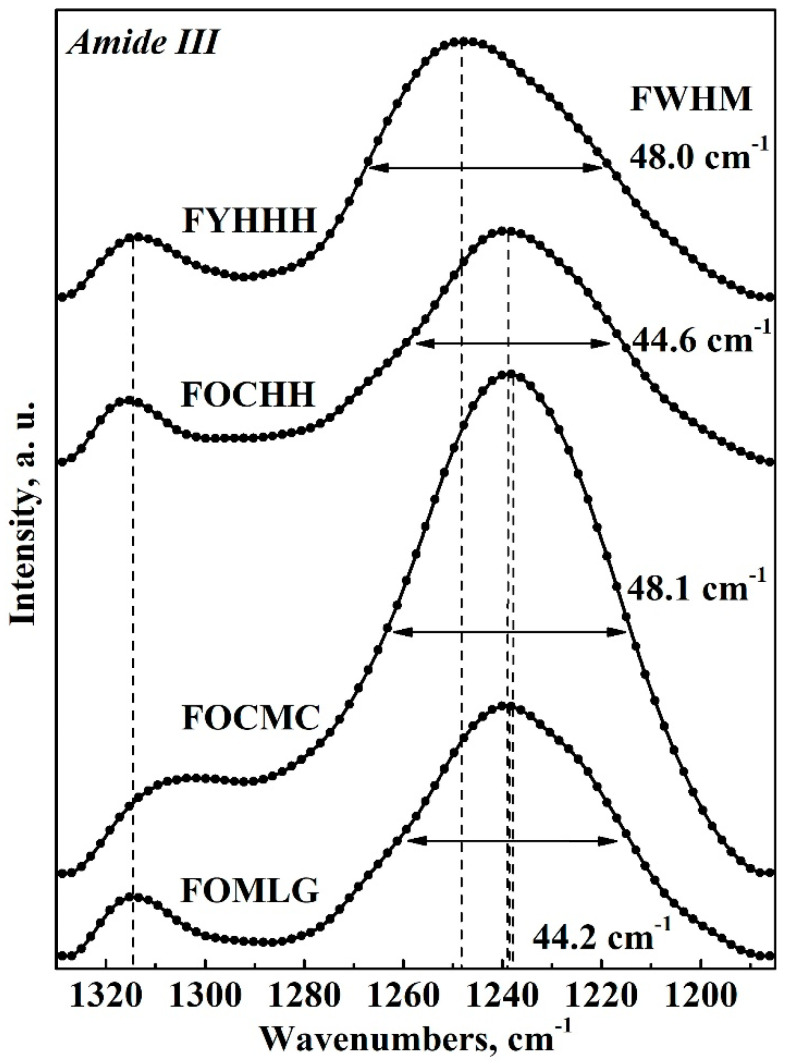
Characteristic spectral profiles in the Amide III band region for selected GCF samples.

Figure 3 presents a selection of FTIR spectra for GCF samples, chosen based on the preliminary results of the multivariate analysis. The spectra are shown within the wavenumber range corresponding to Amide III (1330 to 1190 cm⁻^1^). It is important to note that Figure 3 presents the averaged spectrum for the respective sample types listed in Table 2.

As noted in Section 3.4, each GCF sample (i.e., the contents of the microcapillary) was divided into ten portions, and an individual FTIR spectrum was acquired for each portion. The spectra in the Amide III band region demonstrated consistent spectral profiles across the portions of each sample. However, there was a slight variation in the intensity of the spectral features, likely reflecting both patient-specific biological variability and experimental conditions. The preliminary use of multivariate analysis methods enabled us to account for this variation in the subsequent analyses of the spectral profiles. As a result, the molecular composition of the GCF samples was characterized based on the average spectral profiles.

An analysis of the spectral profiles of the GCF samples set presented in Figure 3 (corresponding to the respondents listed in Table 2) reveals that both the intensity and spectral position of the Amide III band maxima vary depending on the condition of the oral cavity (i.e., factors “**Caries**” and “**Periodontal diseases**”) as well as the presence of comorbidities. These variations are reflected in the shape of the Amide III spectral profile. Notably, a frequency shift in the primary maxima of the Amide III band is observed, consistent with the findings of previous studies examining the molecular composition of dental biofilms [20,34].

The transformation of the Amide III spectral profile, as observed in Figure 3, is associated with changes in the secondary structure of the proteins that comprise GCF [18,35,36,37,38,39]. To assess the contribution of various elements of the secondary structure of proteins to the overall Amide III spectral profile, a deconvolution operation was performed (refer to details in Section 3.5) on the selected spectral profiles (see Table 2).

**Figure 4:**
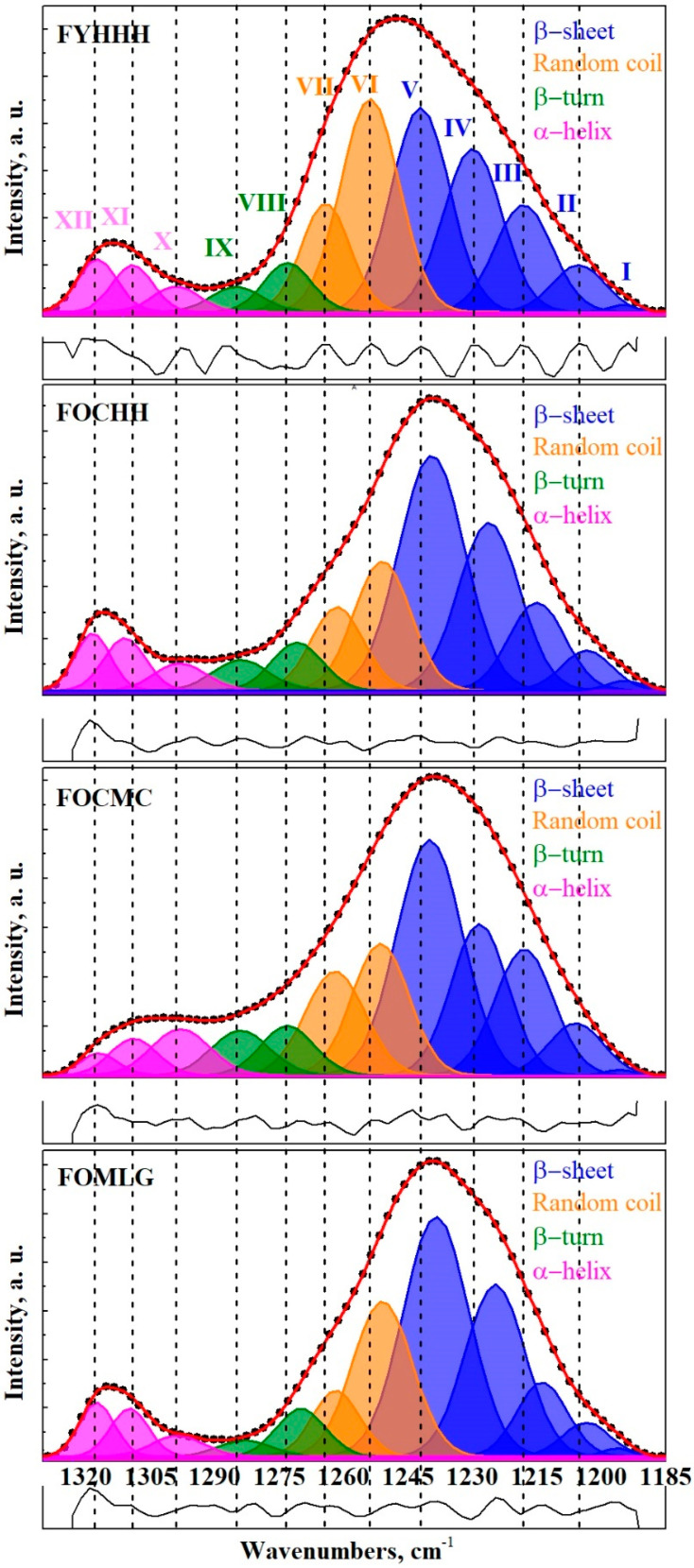
Results of the deconvolution of the spectral profile of the Amide III band of the GCF samples (see Table 2). The absorption bands in the infrared (IR) range can be attributed to the following components of the secondary structure: (XII)—α-helix, 1318.5–1317 cm^−1^; (XI)—α-helix, 1311–1308.8 cm^−1^; (X)—α-helix, 1299–1298 cm^−1^; (IX)—β-turn, 1285–1283 cm^−1^; (VIII)—β-turn, 1273–1270 cm^−1^; (VII)—Random coil, 1264–1261.5 cm^−1^; (VI)—Random coil, 1254–1251 cm^−1^; (V)—β-sheet, 1242–1238.5 cm^−1^; (IV)—β-sheet, 1230–1224.5 cm^−1^; (III)—β-sheet, 1218–1214 cm^−1^; (II)—β-sheet, 1206–1203.5 cm^−1^; (I)—β-sheet, 1196.5–1194.5 cm^−1^.

Figure 4 displays the experimental spectra within the range of 1330–1185 cm⁻^1^ and the results of their deconvolution. The components in the Amide III profile of GCF are labeled with Roman numerals, corresponding to their association with specific secondary structural elements of the proteins.

As a result of decomposing the Amide III spectral band into its components, the maxima that form the spectral profile were associated with the elements of the secondary structure of proteins. Data from several studies were utilized, which examined proteins, including those found in the composition of GCF and oral biofilms [21,22,23,40,41,42,43]. According to the established nomenclature, the following fundamental secondary structural elements are distinguished in the Amide III band profile: regular secondary structures, such as α-helix, β-sheet, β-turn, and amino acid side chains. It is evident that a significant contribution to the integral intensity of the Amide III spectral profiles arises from β-sheets and random coils, which is consistent with the findings of other studies [44]. In contrast, the Amide I and II band profiles show maxima corresponding to structural elements associated with α-helices and β-sheets [20]. Furthermore, it is noteworthy that for all samples, the proportion of β-sheets in the spectral profile significantly exceeds that of the α-helices. A similar observation has been previously reported for proteins during dehydration [45].

The results of the decomposition of the FTIR spectral profiles of the GCF samples into components (including peak frequency, integral intensity, and full width at half maximum (FWHM)), along with their association with the secondary structural elements of proteins, are presented in Table 3.

**Figure 5:**
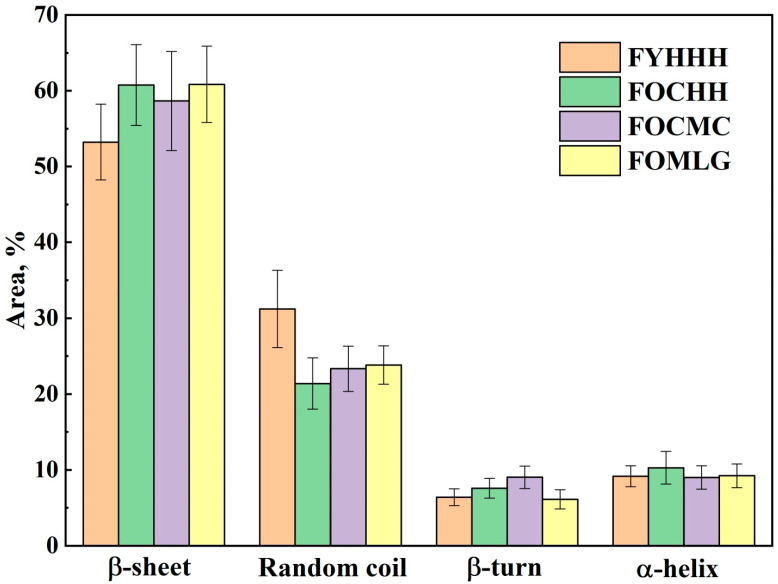
Total contributions of specific secondary structural elements of GCF proteins to the Amide III spectral profile.

Figure 5 illustrates the variations in the total contributions of specific secondary structural elements of GCF proteins to the Amide III spectral profile across different stages of caries and periodontal pathologies, taking comorbidities into account.

The transformation in the contributions of the secondary structural elements to the Amide III band profile is clearly visible depending on the combination of the influencing factors. It is noteworthy that during the development of oral cavity pathologies, the total contribution of β-sheet and random coil structures undergoes significant changes, whereas the change in the total contribution of β-turn and α-helix is not as pronounced.

The integral intensity of the mode in the FTIR spectrum is proportional to the concentration of molecular bonds associated with this mode, although the experimental conditions can influence this value. Therefore, in the analysis of GCF samples, an increase in the integral intensity of the Amide III band under specific conditions (influenced by a particular factor) indicates a change in the concentration of specific proteins in the GCF composition that contribute to this spectral region of the Amide III band, reflecting the complex proteomics of GCF. At the same time, the transformation (i.e., redistribution) of intensity among the components shaping the Amide III spectral profile suggests conformational changes in the secondary structure of GCF proteins. Thus, for a more comprehensive assessment, it is preferable to use various ratios between the intensities (contributions) of the elements of the Amide III spectral profile.

**Figure 6:**
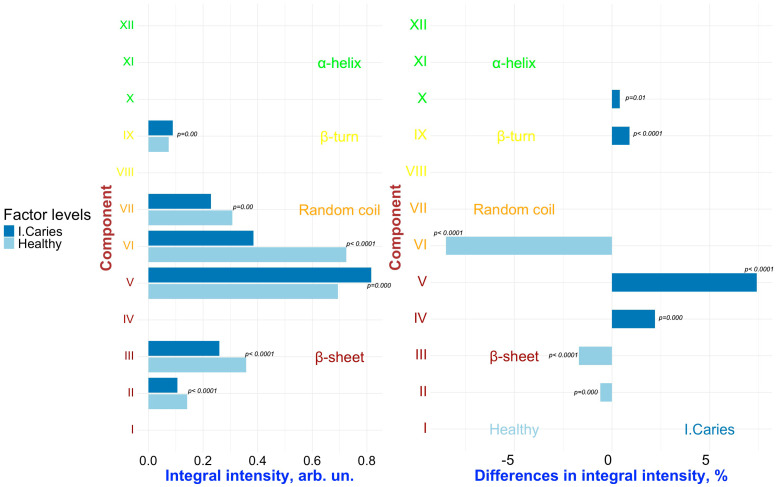
Pairwise comparison of the integral intensity values (**left**) and the change in relative (%) content (**right**) in the averaged spectral profile of the corresponding secondary structural elements for samples FYHHH vs. FOCHH. Analyzed factor: **Caries** (levels: ***Initial Caries*** (I. Caries) and ***Healthy***). Only significant differences are shown.

**Figure 7:**
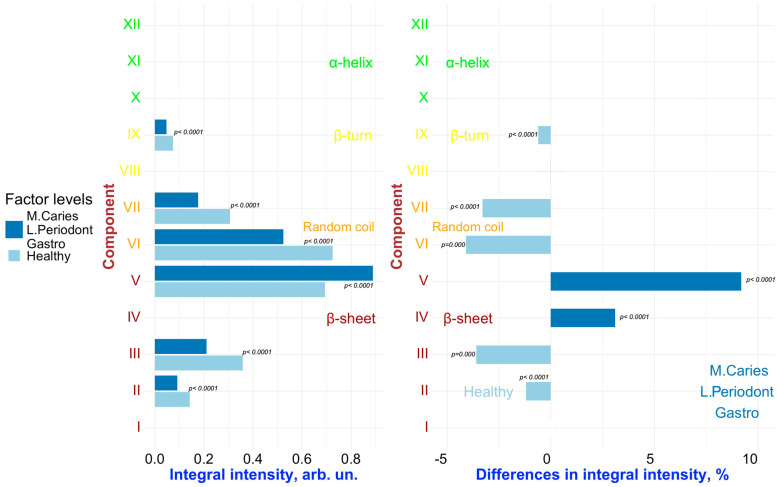
Pairwise comparison of the integral intensity values (**left**) and the change in relative (%) content (**right**) in the averaged spectral profile of the corresponding secondary structural elements for samples FYHHH vs. FOMLG. Analyzed factors: **Caries** (levels: ***Multiple Caries*** (M. Caries), ***Healthy***), **Periodontal diseases** (levels: ***Mild Periodontitis*** (L. Periodont), ***Healthy***), and **Comorbidities** (levels: ***Gastrointestinal Diseases*** (Gastro), ***Healthy***). Only significant differences are shown.

**Figure 8:**
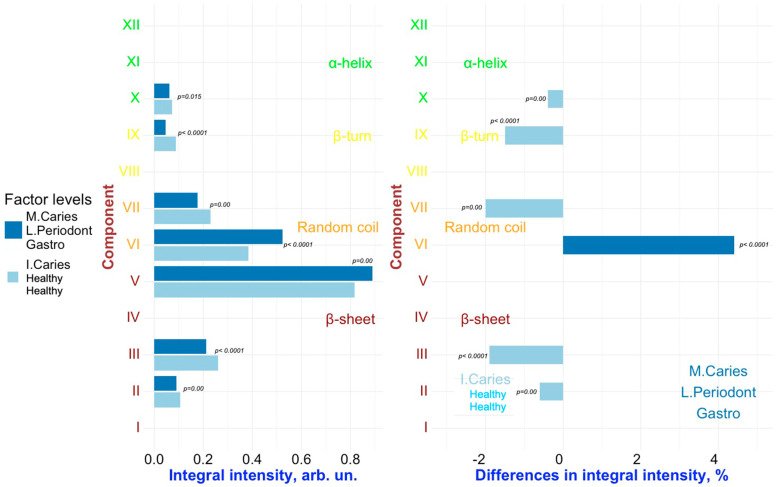
Pairwise comparison of the integral intensity values (**left**) and the change in relative (%) content (**right**) in the averaged spectral profile of the corresponding secondary structural elements for samples FOCHH vs. FOMLG. Analyzed factors: **Caries** (levels: ***Multiple Caries*** (M. Caries), ***Initial Caries*** (I. Caries)), and **Periodontal diseases** (levels: ***Mild Periodontitis*** (L. Periodont), ***Healthy***), **Comorbidities** (levels: ***Gastrointestinal Diseases*** (Gastro), ***Healthy***). Only significant differences are shown.

**Figure 9:**
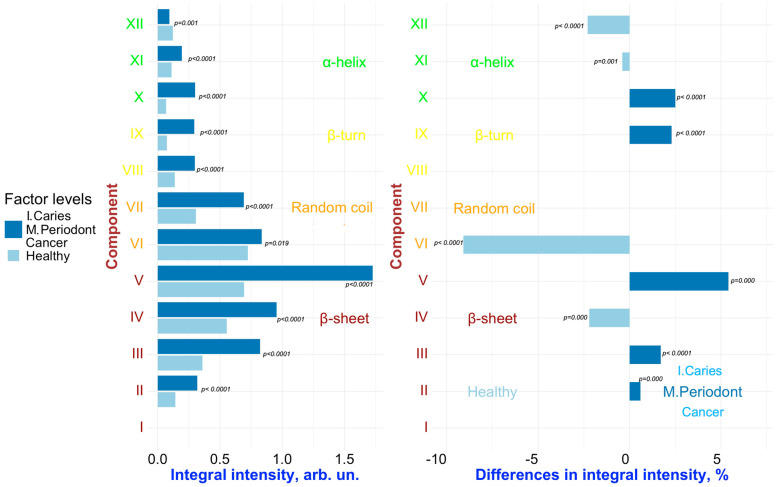
Pairwise comparison of the integral intensity values (**left**) and the change in relative (%) content (**right**) in the averaged spectral profile of the corresponding secondary structural elements for samples FYHHH vs. FOCMC. Analyzed factors: **Caries**, **Periodontal diseases**, **Comorbidities**. Analyzed factors: **Caries** (levels: ***Initial Caries*** (I. Caries), ***healthy***), **Periodontal diseases** (levels: ***Moderate Periodontitis*** (M. Periodont), ***Healthy***), **Comorbidities** (levels: ***Oncological Diseases*** (Cancer), ***Healthy***). Only significant differences are shown.

**Figure 10:**
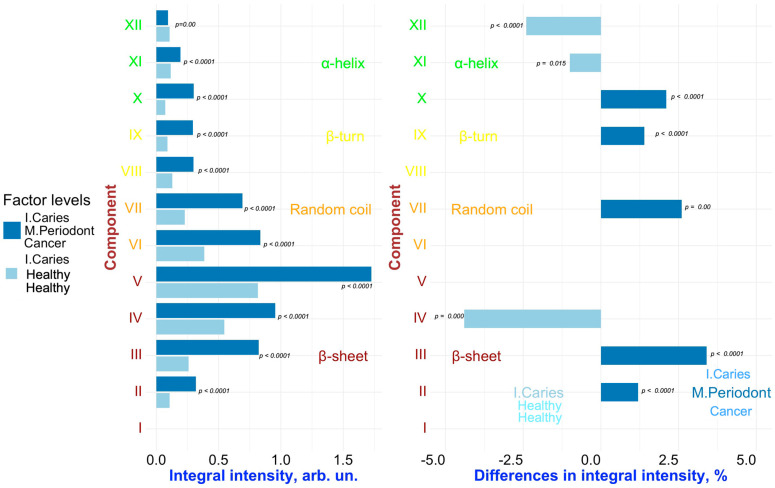
Pairwise comparison of the integral intensity values (**left**) and the change in relative (%) content (**right**) in the averaged spectral profile of the corresponding secondary structural elements for samples FOCHH vs. FOCMC. Analyzed factors: **Periodontal diseases** (levels: ***Moderate Periodontitis*** (M. Periodont), ***Healthy***), **Comorbidities** (levels: ***Oncological Disease*** (Cancer), ***Healthy***). Levels of **Caries** factors for both samples—Initial caries (I. Caries). Only significant differences are shown.

To detect changes associated with specific factors (“**Caries**”, “**Periodontal diseases**”, “**Comorbidities**”), pairwise comparisons were conducted on the results of the deconvolution of the Amide III spectral profiles of GCF samples representing different combinations of these factors. Figure 6, Figure 7, Figure 8, Figure 9 and Figure 10 present the outcomes of these comparisons, highlighting the differences in both the integral intensity values and the relative (%) content of the corresponding secondary structural elements in the averaged spectral profile of the selected samples. Statistical comparisons were performed using the BSDA library with the Bonferroni correction applied to account for multiple comparisons, ensuring the validity of the significance levels for the observed differences (see Section 3).

Analyzing the relationships presented in Figure 6, Figure 7, Figure 8, Figure 9 and Figure 10 (left), it is evident that, despite the significant qualitative differences in the integral intensity of the secondary structural components of GCF proteins, these changes—depending on the influencing factor—rarely exceed 0.2 arbitrary units (arb.un.). This suggests minor quantitative shifts in the protein profile of GCF under the influence of factors associated with the development of oral diseases such as caries and periodontal disease. In contrast, a more pronounced change (ranging from 0.4 to 1 arb.un.) in the integral intensity of secondary structure elements III to V, particularly associated with β-sheets, occurs under the influence of the “***Oncological disease***” level of “**Comorbidities**” factor (Figure 9 and Figure 10, left). This indicates the emergence of additional specific proteins with distinct Amide III profiles in the FTIR spectrum.

Significant and statistically reliable changes in the relative (%) content of secondary structural elements of the GCF proteins are observed under the influence of various factors, reflecting alterations in the local spatial conformation. It is clearly visible that when comparing samples FYHHH and FOCHH (obtained from a healthy respondent and a respondent with initial caries, respectively) (Figure 6, right), changes are evident in the relative (%) content of components III–V (β-sheet), VI (random coil), and IX (β-turn) in the averaged spectral profile.

Similarly, a comparative analysis of the results presented in Figure 7 and Figure 8 (right) allows us to identify the combined influence of the factors “**Periodontal diseases**” and “**Comorbidities**” (in particular, the levels of the factors “***Mild periodontitis”/”Moderate periodontitis***” and “***Gastrointestinal diseases***”, respectively) on the spectral characteristics of GCF. In this case, changes in the relative (%) content of the secondary structure elements of GCF proteins are observed for components VI–VII (random coil) and IX (β-turn). However, it can be noted that for components VI (random coil) and IX (β-turn), the influence of the factors “**Caries**” and “**Periodontal diseases**” leads to opposite changes in the relative content of these components in the average spectral profile. For example, for IX (β-turn), the levels “***Initial caries***” and “***Multiple Caries***” of the “**Caries**” factor are associated to an increase in the relative content of this component in the spectral profile, while the levels of the **“Periodontal diseases”** factor related to periodontitis **(“*Mild periodontitis”*** and ***“Moderate periodontitis*”)** lead to a decrease. For component VI (random coil), the opposite situation is observed (Figure 7 and Figure 8, right). As a result, in the case of superposition (simultaneous influence) of the factors “**Caries**” and “**Periodontal diseases**” (see, for example, Figure 7, right), the outcome (influence) will depend on the level of each of the factors.

It is important to note that, in the dataset used for the selected comparison pairs, it is not possible to fully isolate the individual effects of the factors “**Periodontal diseases**” and “**Comorbidities**” (specifically, the levels related to periodontitis and gastrointestinal diseases, respectively). The observed changes in the secondary structure may be influenced by the combined effect of these two factors. Moreover, the well-established comorbidity of periodontal and gastrointestinal diseases [46,47] further complicates the interpretation, making it challenging to determine the distinct impact of each factor on the Amide III spectral profile of GCF.

Regarding the influence of the “***Oncological disease***” level of factor “**Comorbidities**”, statistically significant differences in the relative (%) content were observed for the XII component (Figure 9 and Figure 10, on the right), which corresponds to α-helix. Specifically, a decrease in α-helix contribution was associated with this factor. Notably, Hemendra Ghimire et al. previously reported that during the development of breast cancer, the content of most elements of the secondary structure of proteins in serum, according to FTIR spectroscopy, remained largely unchanged; however, an increase in β-sheet structures and a decrease in α-helix structures were observed [48]. Similar changes—a decrease in α-helix proteins in the composition of the analyte—were also detected by Raman spectroscopy during the progression of rectal cancer [49].

It is important to note that the conformational analysis of GCF proteins revealed consistent differences in the relative percentage of component V across all pairwise comparisons, where the “**Caries**” factor is present in only one of the samples (Figure 6, Figure 7 and Figure 9 on the right). In these cases, only one sample was obtained from each respondent with caries. In contrast, comparisons between GCF samples from individuals of GCF samples from individuals who both had caries did not show statistically significant differences in component V (Figure 8 and Figure 10 on the right). These findings suggest that component V of the Amide III spectral profile of GCF, associated with β-sheet structures, can serve as a statistically significant marker for dental caries, independent of the presence of other comorbidities.

In summary, the following conclusions can be drawn from the results obtained in this study. The FTIR spectra of GCF are complex and challenging to analyze due to the numerous overlapping vibrational bands associated with specific compounds present under certain conditions. However, by employing multivariate analysis and pairwise comparison of the outcomes of the deconvolution of the Amide III profile, it is possible to identify features of the secondary structure of proteins associated with different factors. These features are linked to the transformation of the molecular composition of GCF proteins during the progression of oral pathologies. Changes in the protein fraction of GCF serve as valuable biomarkers not only for periodontitis [13] but also for the development of dental caries, even in the presence of comorbidities. The alterations identified in this study regarding the protein fraction of GCF and the associated conformational changes are consistent with the findings reported in earlier research using conventional spectroscopic methods [3,18,50,51].

## 3. Materials and Methods

### 3.1. Experiment Design

In this study, we analyzed fluid samples from the gingival sulcus collected from patients during our previous research [14] following the established protocol. At the time of the experiment, the participants were not taking antibiotics or medications, smoking, or consuming alcohol.

The collection of GCF was conducted on participants of European descent between the ages of 25 and 60 years, following their clinical examination at the Dental Clinic of Voronezh State Medical University, named after N.N. Burdenko. All procedures were performed in accordance with the guidelines and recommendations of the World Health Organization (WHO).

During the examination, the presence or absence of dental caries, periodontal diseases, and medical history verified through official records from the patient’s personal medical file were documented for each patient. The classification of carious lesions in the participants’ teeth was performed using the International Caries Detection and Assessment System (ICDAS). Based on this classification, the patients were divided into the following groups: healthy (H, ICDAS 0), initial caries (C, ICDAS 1–2), and multiple caries (M, ICDAS 3–4). The condition of the periodontium was classified according to the system recommended by the Centers for Disease Control and Prevention (CDC) and the American Academy of Periodontology (AAP) for the classification of periodontitis. Participants were assigned to the following groups: healthy periodontal condition (H), mild periodontitis (we used L for this stage of periodontitis to avoid confusion with moderate periodontitis**),** and moderate periodontitis (M). Based on their medical history, patients were classified as healthy (H) or as having comorbidities: gastrointestinal (G), oncological (C), or multiple diseases (M). Additionally, the demographic characteristics of the respondents were recorded, including sex (male—M, female—F) and age, categorized according to the World Health Organization (WHO) classification: individuals younger than 44 years were assigned to the younger age group (Y), and those older than 44 years were assigned to the older age group (O).

### 3.2. Dataset Description

Table 4 presents information about the participants in the study who provided GCF using the notations introduced earlier (see Section 3.1). Samples chosen for detailed analysis are highlighted in bold.

### 3.3. Collection of Gingival Crevicular Fluid

GCF was collected without stimulation during the late morning (10 AM–12 PM) to minimize the effects of the circadian rhythm. The collection was performed using microcapillaries [50,52] with a diameter of 250 μm, which were filled with a homogenized fine powder of potassium bromide (KBr) and compacted with a non-woven filter. The microcapillary was connected to a vacuum pump to enhance the adsorption of GCF by the KBr powder.

Initially, the participants cleaned their oral cavities using a toothbrush and rinsed with clean water. Thirty minutes after the cleaning procedure and after the oral cavity was dried with air, GCF was collected. To isolate the collection site, sterile cotton rolls were placed around the patient’s teeth on both vestibular and oral sides.

The collection time was not fixed, as the rate of GCF release can vary by approximately tenfold depending on the presence or absence of pathology and the extent of periodontal tissue inflammation [53].

After collection, the microcapillaries were promptly placed in sterile containers and stored at a temperature of 1–2 °C.

### 3.4. FTIR Spectroscopy

FTIR spectra were collected using equipment from the Infrared Synchrotron Radiation Beamline at the Pohang Accelerator Laboratory (PAL) in Korea and the Brazilian Synchrotron Light Laboratory. This setup enabled high-precision measurements of the molecular composition of gas chromatography fractions (GCF) collected in microliter volumes. A Bruker Vertex 80/v vacuum FTIR spectrometer (Bruker Optik GmbH, Ettlingen, Germany), equipped with a MIRacle™ Single Reflection ATR attachment featuring a zinc selenide (ZnSe) crystal (Pike, Madison, WI, USA), along with an Agilent Cary 660 FTIR microscope (Agilent Technologies, Santa Clara, CA, USA) with thermal source as illumination, were utilized for the measurements.

FTIR spectra were recorded in the spectral range of 4000–500 cm⁻^1^ with a resolution of 4 cm⁻^1^. Each sample (the contents of the microcapillary) was divided into ten parts, and a separate FTIR spectrum was obtained for each part. The standard parameters for recording the FTIR spectra included Blackman-Harris 3-Term apodization, Mertz phase correction, and a zero-filling factor of 2. The initial processing of the collected spectra was performed using OriginPro 2022.

### 3.5. Spectra Processing

The standard initial processing of the FTIR spectra for GCF involved smoothing the spectral curves using a second-order Savitzky–Golay polynomial function with a window size of 25 points. Baseline correction was performed using the OriginPro 2022 software package (Origin Lab, Northampton, MA, USA), employing the asymmetric least squares method for data in the range of 4000–500 cm⁻^1^, with a threshold value of 0.01, and an asymmetry factor of approximately 0.001.

To determine the contributions of various components of the secondary structure in the Amide III band region, we deconvoluted the FTIR spectral profile. This method enables the assessment of parameters associated with the individual structural elements of the spectral band, including the peak center of gravity, full width at half maximum (FWHM), and integral intensity. Gaussian functions were used to approximate the Fourier transform infrared (FTIR) profile, as they most accurately represent the shape of the modes in the FTIR spectra. Preliminary searches for the centers of gravity and FWHM of the peaks forming the spectral profile were conducted by calculating the second and fourth derivatives, followed by curve smoothing using the Savitsky–Golay polynomial method. Further curve modeling was performed using the Levenberg–Marquardt algorithm implemented in the Fityk v.1.3.1 software package. Fityk is an open-source software developed by Marcin Wojdyr for nonlinear fitting of analytical functions (especially peak-shaped) to data (usually experimental data). To accurately assess the influence of each element of the secondary structure in the Amide III region, we maintained the same number of maxima for each sample. After the preliminary determination of the vibrational mode parameters, the experimental curve was modeled using box constraints. This approach allowed the parameters of the spectral maxima to vary only within a specified, limited range. For the centers of gravity, the constraints were set at ±2 cm⁻^1^, while the acceptable range of values for the FWHM was between 7 and 10 cm⁻^1^. The approximation process was concluded when the best fit between the theoretical and experimental curves was achieved, as determined by the chi-square (χ^2^) criterion. The established convergence and reproducibility criteria d enabled unambiguous decomposition, which was verified using a large set of experimental spectra.

### 3.6. Methods of Data Analysis

After preliminary processing, the array of spectral data was analyzed using principal component analysis (PCA) to assess the proximity of samples within a multidimensional space and explore the relationships between components and factors (categorical variables representing the characteristics of respondents). The data were organized in a matrix format, where the columns represented intensity values across a specified range of wave numbers (corresponding to the spectral profile of the Amide III band) and the rows represented the samples under investigation.

The spectral intensities at specific wavenumber were used as quantitative features (variables). The characteristics of the experimental participants, including demographic information, presence or absence of periodontal diseases, and carious lesions, are presented as categorical variables with varying numbers of factor levels (Table 1). Sex and age were treated as binary factors, while other participant characteristics related to dental status and medical history were represented as multi-category factor variables. Although these categorical variables were not included in the analysis, they were instrumental in interpreting the results.

For interpretation, the initial PCA components were selected, and the relationships between these components and the factors studied were analyzed. At this stage of the analysis, we used the FactoMineR package in R [54]. During the multivariate analysis, the entire spectral dataset (covering all samples) was processed to reveal the underlying structure, including groupings and key factors of variation for further interpretation. FactoMineR allows the establishment of connections between principal components (PCs) and the studied categorical variables using the *dimdesc* function (refer to [55] for a detailed description of the function’s operation). The procedure involves conducting a series of ANOVA tests for each categorical variable across all PCs, yielding F-statistics, R^2^ values, and *p*-values. Following this, a set of *t*-tests is performed for each level of the categorical variables (factors), with the output including estimates of the mean differences and their corresponding *p*-values.

The v. test value was calculated for factors that were significantly associated with the PCs. This statistic indicates the strength and direction of the relationship between the factor level and the intensity values. The *v.test* is included in the output of the *catdes* function of the FactoMineR package.

In addition, we used the *dist* function from the FactoMineR package. This function enables the calculation of the Euclidean distances between samples in the PCA space based on their coordinates in the low-dimensional representation. This allows for a comprehensive quantitative assessment of the proximity between samples in multidimensional space across all studied PCs. The distance matrix generated by this function is employed for Hierarchical Clustering on Principal Components and for elucidating the differences between samples. This function computes the Euclidean distances between samples based on their positions in the PCA space, which reflect the underlying factors and their respective levels.

To visualize the results obtained using the FactoMineR package, we employed the factoextra package [56].

The multivariate analysis of spectral data described herein has already been successfully tested in our previous research [14].

In the next stage, after identifying the key factors contributing to variability in the data, samples were selected for further deconvolution of their spectral profiles. The results of deconvolution were subjected to pairwise comparisons. For these comparisons, we utilized the R package BSDA [57], specifically its function *tsum.test*, which facilitates a two-sided standard two-sample *t*-test. The uniqueness of this library lies in its capability to conduct tests aimed at identifying statistically significant differences between groups based on the means and standard deviations of the integral intensity values for each component of secondary structure. As some samples were used for comparison in more than one pair, we applied the Bonferroni correction for multiple comparisons. This method was selected for its conservative nature, ensuring that significant differences are not overlooked, which aligns with the objective of our study to identify the most meaningful differences between samples. The Bonferroni method is known to be stricter than the Tukey test, which permits type I errors but is less stringent than the very conservative Scheffé method [58]. This test was employed to perform pairwise comparisons between two samples for each parameter (component of the secondary structure) separately, using a 95% confidence interval. The ggplot2 package was used to visualize the results of pairwise comparisons [59].

## 4. Conclusions

This study is the first to apply multivariate data analysis to deconvolute the spectral profiles of Amide III in the FTIR spectra of gingival crevicular fluid (GCF). This demonstrates how major oral diseases, such as dental caries and periodontal diseases, along with various comorbidities, affect the transformation of the secondary structure of GCF proteins. A two-stage analytical approach was employed: initially, principal component analysis (PCA) was used to establish the primary sources of variation in the spectral data, followed by pairwise comparisons of the outcomes of Amide III profile deconvolution to evaluate the specific effects of individual factors. The methodology described in this study was effective in identifying potential biomarkers in GCF.

For the first time, we demonstrated that the relative content of the β-sheet-associated component in the spectral profile of the protein fraction of GCF serves as a statistically significant marker for dental caries, regardless of the presence or absence of other comorbidities. Additionally, a statistically significant decrease in the relative content of α-helix structures in the protein fraction of GCF was observed in patients with oncological diseases.

The alterations in the spectral profile of the Amide III band of GCF discussed in this study have not been previously identified through molecular spectroscopy, nor have they been correlated with the secondary structure of proteins or examined using multivariate data analysis techniques.

The characteristics of secondary protein structure transformations in GCF identified in this study may be utilized for expedited screening and monitoring of dental caries and periodontal diseases at a new level of precision diagnostics, taking into account patient comorbidities. The use of GCF is a relatively rapid and minimally invasive diagnostic method, and when combined with the FTIR express analysis technique, it provides an innovative approach for delivering personalized dental care.

### Limitations

A significant limitation of this study is the small sample size and the presence of infrequently occurring factors within the individual respondents. This combination hindered the independent assessment of the influence of periodontal disease and gastrointestinal pathologies due to their comorbidity.

The limitation associated with the small sample size arises from the labor-intensive procedures involved in the sample extraction and preparation. However, by employing a combination of various multivariate data analysis methods tailored for spectral data, including techniques specifically designed for small samples, we were able to mitigate these inherent limitations to the greatest extent possible.

## Figures and Tables

**Table 1:** Euclidean distances between samples based on factor levels.

Factor (Categorical Variable)	Factor Levels with Corresponding Distance Values			
Gender	Female	Male		
	1.08	3.53		
Age	Old	Young		
	0.7	0.6		
Caries	Healthy	Initial	Multiple	
	2.8	0.6	2.7	
Periodontal **diseases**	Healthy	Mild periodontitis	Moderate periodontitis	
	0.4	2.65	**15.42**	
Comorbidities	Oncological disease	Gastrointestinal diseases	Healthy	Multiple pathologies
	**5.47**	2.89	1.7	2.85

**Table 2:** Characteristics of respondents whose GCF samples were selected for Amide III spectral profile deconvolution.

Participant Identifier	Gender	Age	Caries	Periodontal Diseases	Comorbidities
FYHHH	F	Y	H	H	H
FOCHH	F	O	C	H	H
FOCMC	F	O	C	M	C
FOMLG	F	O	M	L	G

**Table 3:** Results of the deconvolution of the Amide III region of the GCF FTIR spectral profiles into components (including peak frequency, integral intensity, and FWHM) and their association with the secondary structural elements of proteins.

Component	Assignment [21,22,23,40,41,42,43]	FYHHH	FOCHH	FOCMC	FOMLG
		Wavenumber, cm^−1^ Integ. Int., % FWHM, cm^−1^	Wavenumber, cm^−1^ Integ. Int., % FWHM, cm^−1^	Wavenumber, cm^−1^ Integ. Int., % FWHM, cm^−1^	Wavenumber, cm^−1^ Integ. Int., % FWHM, cm^−1^
XII	α-helix	1317.3 (±0.7)	1318.4 (±0.9)	1317.0 (±0.9)	1317.5 (±0.8)
		3.7 (±0.60)	3.8 (±0.35)	1.4 (±0.15	3.6 (±0.68)
		7.4 (±0.5)	9.2 (±0.3)	8.0 (±0.4)	8.1 (±0.4)
XI	α-helix	1309.2 (±0.6)	1310.9 (±0.9)	1308.8 (±0.7)	1309.8 (±0.8)
		3.4 (±0.30)	4.0 (±1.05)	3.0 (±0.31)	3.5 (±0.34)
		14.3 (±0.4)	13.7 (±0.5)	15.0 (±0.3)	13.0 (±0.6)
X	α-helix	1299.0 (±0.7)	1298.1 (±0.6)	1298.0 (±0.7)	1298.6 (±0.6)
		2.1 (±0.30)	2.5 (±0.35)	4.6 (±0.76)	2.1 (±0.34)
		15.7 (±0.5)	15.3 (±0.6)	16.0 (±0.3)	13.9 (±0.6)
IX	β-turn	1285.0 (±0.9)	1283.9 (±1.0)	1284.0 (±0.6)	1283.0 (±1.1)
		2.2 (±0.30)	3.1 (±0.35)	4.5 (±0.61)	1.6 (±0.34)
		16.0 (±0.6)	16.8 (±0.5)	15.5 (±0.7)	16.5 (±0.4)
VIII	β-turn	1273.0 (±0.8)	1270.8 (±0.9)	1273.0 (±0.6)	1269.9 (±0.6)
		4.2 (±0.60)	4.5 (±0.70)	4.6 (±0.76)	4.5 (±0.68)
		16.0 (±0.5)	18.0 (±0.5)	18.0 (±0.6)	18.0 (±0.5)
VII	Random coil	1264.2 (±0.7)	1261.5 (±0.7)	1262.0 (±0.8)	1261.8 (±0.9)
		9.3 (±1.51)	8.0 (±1.74)	10.6 (±1.22)	6.0 (±0.68)
		16.0 (±0.4)	15.4 (±0.5)	15.6 (±0.6)	16.4 (±0.6)
VI	Random coil	1253.9 (±0.8)	1251.0 (±1.0)	1251.5 (±0.6)	1251.0 (±0.9)
		21.9 (±2.72)	13.4 (±1.39)	12.8 (±1.53)	17.8 (±1.36)
		13.3 (±0.5)	14.2 (±0.7)	16.3 (±0.6)	13.0 (±0.7)
V	β-sheet	1242.1 (±0.9)	1239.5 (±0.6)	1240.0 (±1.1)	1238.5 (±0.7)
		21.0 (±2.12)	28.4 (±2.09)	26.4 (±3.06)	30.2 (±1.70)
		13.1 (±0.5)	13.9 (±0.4)	14.8 (±0.3)	13.4 (±0.5)
IV	β-sheet	1230.0 (±0.7)	1226.2 (±0.8)	1228.4 (±0.7)	1224.7 (±0.6)
		16.8 (±0.91)	19.0 (±1.39)	14.6 (±1.22)	19.9 (±1.36)
		14.0 (±0.6)	15.2 (±0.5)	16.0 (±0.6)	14.3 (±0.6)
III	β-sheet	1218.1 (±0.9)	1215.0 (±1.0)	1218.0 (±0.6)	1213.8 (±0.8)
		10.8 (±0.60)	9.1 (±0.35)	12.5 (±0.61)	7.2 (±0.68)
		12.7 (±0.5)	14.0 (±0.3)	16.0 (±0.5)	14.2 (±0.4)
II	β-sheet	1205.3 (±0.8)	1203.4 (±1.0)	1206.0 (±0.9)	1203.5 (±1.1)
		4.3 (±0.30)	3.7 (±0.35)	4.9 (±0.31)	3.1 (±0.34)
		11.1 (±0.6)	11.3 (±0.7)	12.9 (±0.4)	10.4 (±0.3)
I	β-sheet	1195.0 (±0.9)	1194.5 (±0.7)	1196.0 (±0.8)	1196.4 (±0.8)
		0.3 (±0.30)	0.6 (±0.35)	0.3 (±0.15)	0.5 (±0.34)
		11.0 (±0.7)	9.8 (±0.5)	10.5 (±0.6)	9.6 (±0.5)

**Table 4:** Information about participants: gender: F—female, M—male; age: Y—young (25–44 years), O—old (45–60 years); caries: H—healthy, C—initial caries, M—multiple caries; periodontal diseases: H—healthy, L—mild periodontitis; M—moderate periodontitis; comorbidities: H—healthy, G—gastrointestinal diseases, C—oncological disease, M—multiple pathologies.

Participant Identifier	Gender	Age	Caries	Periodontal Diseases	Comorbidities
FYHHH	F	Y	H	H	H
**FYCHH**	**F**	**Y**	**C**	**H**	**H**
FYCHH	F	Y	C	H	H
**FOCHH**	F	O	C	H	H
MYCLG	M	Y	C	L	G
MOCLH	M	O	C	L	H
**FOCMC**	F	O	C	M	C
FYCHG	F	Y	C	H	G
MYCLH	M	Y	C	L	H
FYCHM	F	Y	C	H	M
**FOMLG**	F	O	M	L	G
MOMLG	**M**	**O**	**M**	**L**	**G**
FOMHC	F	O	M	H	C
FOMLM	F	O	M	L	M
FOMLG	F	O	M	L	G
FYMLM	F	Y	M	L	M
FYCHH	F	Y	C	H	H

## Data Availability

The data that support the findings of this study are available from the corresponding author upon reasonable request.

## Abbreviations

GCF	gingival crevicular fluid
FTIR	Fourier Transform Infrared
PCA	principal component analysis
